# Examining Social Media Crisis Communication during Early COVID-19 from Public Health and News Media for Quality, Content, and Corresponding Public Sentiment

**DOI:** 10.3390/ijerph18157986

**Published:** 2021-07-28

**Authors:** Melissa MacKay, Taylor Colangeli, Daniel Gillis, Jennifer McWhirter, Andrew Papadopoulos

**Affiliations:** 1 Department of Population Medicine, University of Guelph, Guelph, ON N1G2W1, Canada; colangeli.taylor@gmail.com (T.C.); j.mcwhirter@uoguelph.ca (J.M.); apapadop@uoguelph.ca (A.P.); 2 School of Computer Science, University of Guelph, Guelph, ON N1G2W1, Canada; dgillis@uoguelph.ca

**Keywords:** crisis communication, risk communication, social media, Facebook, COVID-19, public health, pandemic, digital communication, sentiment analysis

## Abstract

Rising COVID-19 cases in Canada in early 2021, coupled with pervasive mis- and disinformation, demonstrate the critical relationship between effective crisis communication, trust, and risk protective measure adherence by the public. Trust in crisis communication is affected by the communication’s characteristics including transparency, timeliness, empathy, and clarity, as well as the source and communication channels used. Crisis communication occurs in a rhetorical arena where various actors, including public health, news media, and the public, are co-producing and responding to messages. Rhetorical arenas must be monitored to assess the acceptance of messaging. The quality and content of Canadian public health and news media crisis communication on Facebook were evaluated to understand the use of key guiding principles of effective crisis communication, the focus of the communication, and subsequent public emotional response to included posts. Four hundred and thirty-eight posts and 26,774 anonymized comments were collected and analyzed. Overall, the guiding principles for effective crisis communication were inconsistently applied and combined. A limited combination of guiding principles, especially those that demonstrate trustworthiness, was likely driving the negative sentiment uncovered in the comments. Public health and news media should use the guiding principles consistently to increase positive sentiment and build trust among followers.

## 1. Introduction

Despite public health’s ability to rapidly detect and respond to emergencies, communication regarding these crises and their recommended measures remains a significant challenge [1,2]. The COVID-19 pandemic has demonstrated the critical requirement of effective crisis communication, with infectious disease spread heavily relying on the public following risk protective measures. In January 2021, many countries, including Canada, experienced a record-breaking number of COVID-19 infections [3]. The lack of adherence to public health restrictions in Canada may, in part, be due to the lack of clarity and transparency in communication from officials including government and public health [4]. Additionally, effective crisis communication should be delivered in many ways, across multiple channels, and must reflect the public’s needs [5]. Ineffective communications and inconsistent and conflicting messages between actors during COVID-19 have been reported, which can impact on belief in misinformation, perceived risk, and appropriate response by the public [6,7,8].

Among the key actors that can influence the trajectory of a crisis are public health officials and news media. Public health, the complex network of organizations promoting and protecting health, has a key responsibility for effective media communication during a public health emergency [1]. News media plays a critical role, providing accurate and factual information about the risks to the public as well as reporting on the activities of government and organizations to ensure accountability [9]. Public health and the media have an interdependent relationship during public health crises, where the media rely on public health for timely, accurate information, and public health relies on the media for amplification of messages [2]. Public health must understand the role of the media within a public health crisis and must employ effective crisis communication techniques to ensure they are providing the media with the information they want and need, ensuring the public will be informed, be able to act upon recommendations, and ultimately maintain trust in crisis communications [9].

Trust plays a pivotal role in the adoption of risk protective behaviours during a pandemic. Trust and communication are intricately linked in pandemics; trust can affect the perception of communication, while communication can build or erode trust [10,11,12]. Trust is highly complex and relational [13] and is mediated by trustworthiness, which is a characteristic of a trusted organization and is influenced directly by the strategies and actions of an organization [14]. Communication contributes to trustworthiness due to the fact of its critical role in relationships [14]. Message characteristics, including the quality, content, and communication channels used to disseminate messages play an important role in influencing risk perception and trust [15]. Public health’s crisis communication should incorporate the characteristics of messages that can demonstrate trustworthiness in order to build trust and positively influence adherence to public health measures.

Crisis communication occurs in a rhetorical arena where actors, including the public and news media, are co-producing, and responding to messaging [2,16,17]. Within the crisis arena, actors are creating and responding to official messages and can influence the interpretation of these messages across many different communication channels [16,17]. Official crisis communication actors, such as public health, must monitor the arenas to understand reactions to their efforts, as these reactions provide assessments of communication effectiveness [7,16]. Social media channels create crisis sub-arenas where various actors are co-creating messaging [17], and crisis actors need to understand how the public is reacting to their efforts in these various sub-arenas [16]. Within social media sub-arenas, messages posted by the public can be analyzed to assess whether they accept the messages and if they are acting upon recommendations [16,17]. Social media provides an important platform that should be used, in conjunction with other communication strategies, for two-way communication with the public as well as to provide essential information about risks, allowing individuals to share evidence-based information with their networks [18].

Facebook is the most widely used social media platform in Canada with 14 million daily users in Canada [19], and many public health, governmental, and news media organizations use it to communicate with the public. In Canada, 83% of adults have a Facebook account, with women more likely than men to have an account and those with the least education or not employed the least likely to have an account [20]. In terms of monthly active users, the most dominant age group on Facebook is 25–34 (89%) year olds, followed by 45–55 (83%), 35–44 (82%), 18–24 (77%), and 55 plus (72%) year olds [20]. Moreover, Facebook is the leading social network used by internet users in all provinces across Canada [21], and it is the most popular source of COVID-19 information among those that have a social media account [22]. The response to official crisis communication within a social media sub-arena, such as Facebook, can be evaluated through examining the public’s emotional reaction [16] to the content via comments. Sentiment analysis allows for a snapshot of emotional response to crisis communication [23], and it has been used during the COVID-19 pandemic to evaluate public response to messaging [24,25].

An evaluation of Canadian public health and news media Facebook posts in relation to key guiding principles for effective crisis communication has not be completed during the COVID-19 pandemic. Evaluation of crisis communication during the first wave is critical for the application of findings and adaptation in order to increase the effectiveness during subsequent waves. As COVID-19 vaccines are being rolled out across Canada, a coordinated and effective communication strategy is necessary to overcome widespread vaccine hesitancy and continued adherence to risk protective measures [26].

Although the news media serves a different purpose compared to public health in terms of crisis communication, evaluating their messaging for quality and content reflects whether they are receiving the information they want and need from public health to amplify effective messages. Furthermore, evaluating the impact of the guiding principles on the public’s emotional response to messaging provides public health and news media with insight into the acceptance and impact of their crisis communication that they can then apply to increase the effectiveness of their efforts.

This research aimed to evaluate the quality and content of Canadian public health and news media crisis communication during the first wave of the COVID-19 pandemic on Facebook and the subsequent emotional response to messaging by the public. The objectives of this research were to:

1. Develop a succinct list of guiding principles for crisis communication on social media using existing literature to maintain trust in order to evaluate the quality of public health and news media messages;
2. Analyze whether and how Canadian public health and news media are incorporating these guiding principles into their COVID-19 crisis communication on Facebook;
3. Determine the topic(s) of COVID-19 crisis communication on Facebook including the situation, resources, and actions to promote and protect health during the pandemic;
4. Understand public sentiment within comments in relationship to the guiding principles and topics included in COVID-19 crisis communication on Facebook;
5. Examine the relationship between the guiding principles used in COVID-19 crisis communication Facebook posts and the corresponding sentiment by source (public health, news media).

## 2. Materials and Methods

### 2.1. Ethics

As per the University of Guelph’s Research Ethics Board, this study did not require ethics approval as the data were collected from publicly available Facebook pages. The collected comments from the included Facebook posts were anonymized to protect the privacy of individuals and were not used as examples to illustrate the sentiment analysis in this research.

### 2.2. Guiding Principles

A literature search was conducted using Google Scholar and Omni databases to find relevant journal articles regarding guiding principles and best practices for crisis communication on social media. There are various resources for crisis communication during emerging infectious diseases crises; however, these do not necessarily include social media crisis communication best practices in an easy-to-implement approach. Appropriate combinations of the following terms were used: health communication, risk communication, crisis communication, pandemic, public health emergency, social media, guidelines, best practices, and guiding principles. Google was used to identify relevant grey literature using the same search terms, in addition to searching known, specific public health websites such as the WHO, CDC, and the Public Health Agency of Canada (PHAC). Reference lists from identified grey literature and review articles were used to identify potential additional sources. Relevant references were added to an Excel spreadsheet, where definitions of best practices and guiding principles were compared and contrasted to develop the list of five guiding principles relevant for crisis communication to maintain trust and two social media best practices that are described in Table 1 in Results.

### 2.3. Data Collection

The research team collected COVID-19-related Facebook posts in June 2020 from three Facebook pages representing Canadian federal public health and national news media: Healthy Canadians, CTV News, and CBC News. At the time of writing, Healthy Canadians was the sole federal public health Facebook page with 366,200 followers [27]. Similarly, CBC News and CTV News are two of the most popular and trusted national news sources among Canadians [25], with 2,746,966 and 996,977 Facebook followers, respectively [28,29]. The three sources were chosen to analyze public health and news media at the national level in order to analyze macrolevel crisis communication.

Facebook’s advanced search function was used to collect posts, including text and graphic content, from the three pages, with two searches being run on each page: “COVID and coronavirus” and “masks and social distance”. Posts made between 31 December 2019 (i.e., first case of pneumonia without a known cause identified) and 14 June 2020 (i.e., day prior to data collection) were included and sorted by ‘top posts’ within the advanced search function to collect posts with the highest engagement. Additional inclusion criteria for posts were English language, written directly by the source, and posts relevant to the provincial or national scope.

Comments on included posts were collected and anonymized. Inclusion criteria for comments were English language; text-based; appeared to be written by a real person by examining the user profile (absence of real photo, followers, or biography), reply syntax (repetitive or formulaic), and semantics (reposting the same reply over and over) (Knight, 2018). Names within comments were removed unless it was a public figure’s name.

Three researchers manually collected the data, which is a common method of capturing publicly available Facebook posts for content analysis [30]. An identification number was created for each post, and the post content, article link, video link, date posted, date collected, and the number of reactions, comments, and shares were collected and input into an Excel spreadsheet as well as a screenshot of each post into a shared drive. Reactions and shares were beyond the scope of this research, but photos and videos were analyzed for clarity (i.e., whether the graphic or video enhanced understanding of the post content).

### 2.4. Content Analysis

#### 2.4.1. Guiding Principles and Topics

As described in the results section, seven guiding principles—five key to crisis communication and trust and two social media best practices—were used to assess the quality of crisis communication on Facebook. For a post to employ one of the guiding principles, it had to include at least one of the guiding principle’s key features noted in Table 1. When actors communicate risk and crisis-related messages, they should include content about the situation (e.g., number of cases), resources (e.g., infographics), and actions (e.g., a direct request to wear a mask) the public should take during a public health emergency. To assess the content of the posts, the three topics (i.e., situation, resource, and action) were identified to classify the focus of the crisis communication messages (Table 2).

Two researchers independently coded all included Facebook posts for the seven guiding principles (Table 1) and three topics (Table 2) using NVivo 12 Plus. Pre-testing for coding was completed until a kappa > 0.8 was achieved for inter-coder reliability, and independent coding proceeded. Disagreements were discussed and consensus was built collaboratively.

#### 2.4.2. Sentiment Analysis

SentiStrength (Java version) was used to estimate the sentiment of Facebook users’ comments, as this program is specifically designed to assess informal pieces of text and has been used in similar research [31]. SentiStrength was used to run a trinary sentiment analysis, classifying each comment as positive, negative, or neutral. The program does this by assigning each word a numerical sentiment score on a scale of positive (+1 not positive to +5 extremely positive) to negative (−1 not negative to −5 extremely negative) [32]. Next, the program determines the difference between the most positive and most negative word in the text to provide an overall trinary sentiment score [32]. For example, the hypothetical comment, “Make sure you physical distance when you are around other people”, has an overall trinary result of neutral or 0. The maximum positive value for this sentence is +1, and the maximum negative value is −1, resulting in a difference of 0.

Some of the word’s pre-assigned sentiment scores were modified after testing, as they were inaccurately driving negative sentiment, as is sometimes the case with highly specific events [31]. For example, if a comment said, “I wear a mask to keep myself from getting sick”, SentiStrength would classify it as negative because it assigns the word “sick” with a sentiment strength score of −2, which is greater than the highest positive score in this sentence which is +1. However, in the context of COVID-19, sick is not necessarily a negative but a more neutral term. Therefore, by changing this word’s score to be neutral (−1), there is no difference between the positive and negative scores and, thus, the sentence is now classified as neutral which is more appropriate. Words that had their pre-assigned sentiment scores changed from negative (score of −2 to −5) to neutral (−1) included: “death”, “dying”, “emergency”, “ill”, “infect”, “isolate”, “risk”, and “sick”, as these words were often used to talk about COVID-19 but not always in a negative context (e.g., “self isolation prevents others from getting sick”). The acronym and idiom lists were also modified to include those that frequently appeared in the comments (e.g., “BS” and “shut up”). Finally, the program’s spelling correction list, booster word list, negating word list, emoticon list, and standard settings were applied.

#### 2.4.3. Statistical Analysis

Data were collated so that each post was labeled based on its source (Healthy Canadians, CBC News, or CTV News), type and number of guiding principles used, and topics addressed as well as the number of comments on each post that were classified as having negative, neutral, or positive sentiment. CTV News and CBC News are collectively referred to as “news media” in this paper. Data were aggregated and evaluated using chi square tests to identify differences across sources, guiding principles, and sentiment. Data were analyzed in SPSS 26 (Chicago, IL, USA).

## 3. Results

A total of 438 Facebook posts were collected based on the inclusion criteria: 112 for Healthy Canadians, 157 for CBC News, and 169 for CTV News. We also collected a total of 26,774 anonymized Facebook comments: 2211 from Healthy Canadians, 11,554 from CBC News, and 13,009 from CTV News.

### 3.1. Guiding Principles and Topics

#### 3.1.1. Guiding Principles

Five guiding principles relevant to effective crisis communication that contribute to public trust and two social media best practices can be found in Table 1. Each guiding principle, their key features, and example posts are outlined in Table 1.

**Table 1:** Key features of guiding principles for crisis communication using social media.

Guiding Principle	Key Features	Example Text
Call to Action ^1^	Directly asks the public to do something as a result of the information [33,34,35] e.g., Visit a website, share the post, watch a video, look at an infographic, help others, etc.	To find out more information, click on the link.
Clarity	Uses plain language (i.e., common terms, parallel form, short sentences) [9,36,37] Conveys complex information visually [33] Targets and tailors information to audience(s) [33,36,38]	Parenting during a pandemic can be stressful. Take a moment for yourself.
Compassion and Empathy	Validates and shows emotion [36] Expresses concern and willingness to impact future tragedy [34,36,38]	After a cold winter and long periods of isolation due to #COVID19, the temptation to get outside is understandable.
Conversational Tone ^1^	Balances friendly conversational tone with professionalism [33,39] Uses first or second person, contractions, and implements good spelling and grammar [33]	Do your part to help prevent #misinformation about #COVID19. Please #thinkbeforeyoushare.
Correction of Misinformation	Addresses and corrects misinformation including rumours and myths [9,10,37,38,40]	Health Canada is warning Canadians about the risks of buying health products, including drugs, natural health products, homeopathic products, and medical devices that make false or misleading claims to prevent, treat, or cure COVID-19.
Timeliness	Communicates information and decisions as they become available or are made [10,41] Shares information within 24 h of first release, based on COVID-19: a timeline of Canada’s first-wave response [42]	Mandatory measures require all travellers entering Canada wear a non-medical mask or face covering during onward travel and avoid contact with vulnerable people. This is in addition to the need to self isolate for 14 days.
Transparency	Provides honest and accurate information [9,35,37] Shares strengths and weakness, uncertainties, and completeness of information [36,37,38] Communicates future research/decisions/how they will go about finding answer [9,40]	At this time, there is no evidence that a mother can transmit #COVID19 to her baby through childbirth, if she gets sick in the third trimester.

^1^ Social media best practice.

#### 3.1.2. Topics

The three topics (i.e., situation, resource, and action), their corresponding features, and example post text are outlined Table 2.

**Table 2:** Key features of crisis communication topics.

Topic	Key Features	Example Text
Situation	Information and knowledge sharing about COVID-19 information, statistics, and epidemiological information	The first reported case of #COVID19 reported in Canada displayed symptoms on 15 January 2020. Since then, the number of cases has increased significantly.
Resource	Tools to assist individuals, communities, or vulnerable populations in protecting and promoting their health	If you think you’ve been exposed to COVID-19, use this self-assessment tool to help you determine if you need further assessment or testing.
Action	Directive request or order to engage in health protective behaviours or actions	Fact check: There is currently no cure for #COVID19. Help us fight misinformation and keep Canada safe. Do not: Share false or unproven information Do: Seek out credible sources

Adapted from Crisis and Emergency Risk Communication: Pandemic Influenza by B. Reynolds (2007), Centers for Disease Prevention and Control. https://emergency.cdc.gov/cerc/resources/pdf/cerc-pandemicflu-oct07.pdf. Accessed on 5 April 2021.

### 3.2. Content Analysis

The use of the guiding principles we identified from the literature (Table 1) in posts by sources varied greatly (Table 3). Call to action was by far the most used guiding principle, with 92–99% of posts including a link to the source’s website. Conversational tone, a best practice for social media communication, was highly used by Healthy Canadians (90% of posts) but only in an average of 26% of news media posts. Timeliness was reflected in an average of 23% of news media posts but only 6% of Healthy Canadians posts. Clarity was used in 21% of Healthy Canadian posts and 16% of CBC and 17% of CTV posts. Compassion, correction of misinformation, and transparency were only used in 5% or less of posts for all sources.

Furthermore, the combination of guiding principles used per post varied by source (Table 4). Healthy Canadians primarily used between 2 (61.61%) and 3 (28.57%) guiding principles per post. This is compared to CBC and CTV News primarily using between 1 (~40%) and 2 (~45%).

Topics incorporated into posts by news media and public health differed (Table 5). News media almost exclusively focused on the situation in their posts (92% average), compared to 26% of Healthy Canadian posts that focused on the same. Healthy Canadians incorporated resources (89% of posts), such as tools that help people learn about and protect their health, and actions (54%) such as direct requests to the reader to engage in preventative behaviours. On average, 16% and 2% of news media posts incorporated resources and actions, respectively.

### 3.3. Sentiment

#### 3.3.1. Pattern of Sentiment by Source

The pattern of sentiment differed across the sources (Table 6) and was statistically significant (*p* < 0.05). Healthy Canadians posts were evenly distributed across the three levels of sentiment, while posts made by news media (i.e., CBC and CTV) were found to invoke approximately 50% more negative comments than positive. That is, for each positive comment CBC or CTV News received on a post, on average, they received 1.5 times more negative comments.

#### 3.3.2. Pattern of Sentiment across the Types of Guiding Principles Used

The pattern of sentiment differed across the guiding principles (Table 7) and was statistically significant for all sources combined (*p* < 0.05).

Among all sources, the guiding principle with the highest negative-comment-to-positive-comment ratio was correction of misinformation. The lowest such ratios were found for conversational tone, timeliness, and call to action. Interestingly, Healthy Canadians posts that were labeled as clarity and correction of misinformation saw fewer negative comments than positive ones. This is compared to the posts by the news media, where all guiding principles had 43–105% more negative comments than positive ones.

#### 3.3.3. Pattern of Sentiment across the Post Topics

The relationship between post topic (Table 8) and the distribution of positive, neutral, and negative comments was found to be significantly different for all sources combined (*p* < 0.05). Approximately 7%, 33%, or 49% more negative comments would be expected if a post was classified as including an action, resource, or situation respectively. Posts labeled as situation were more likely to see the highest negative-to-positive comment ratio. As found with the guiding principles, this pattern did not hold when we considered source. Healthy Canadians’ posts were found to have similar patterns of negative, neutral, and positive comments, regardless of the topic. Posts by the news media, however, saw 34–50% more negative comments.

## 4. Discussion

We analyzed the COVID-19 crisis communication sub-arena on Facebook created by different crisis content producers including public health, news media, and the public. Our analysis addressed whether official crisis communication employed key characteristics that ultimately contribute to maintaining trust and promoting the adoption of risk protective behaviours, as well as the publics’ emotional response to the messages. Our research found that the guiding principles for crisis communication to maintain trust were not consistently applied in Healthy Canadians’, CTV News’, and CBC News’ Facebook posts. Those principles rarely and/or inconsistently applied in communication by all sources included clarity, transparency, compassion and empathy, and timeliness.

### 4.1. Lessons Learned: Public Health Should Consistently Apply the Guiding Principles

Given the limited combination of key guiding principles likely driving negative sentiment, public health officials should consistently apply as many guiding principles as are relevant to the content in social media crisis communication. Typically, Healthy Canadians used between two and three guiding principles per post. The most widely used guiding principles included a call to action (98%) and a conversational tone (90%), which are best practices for social media communication [43]. There were typically more negative comments per post, regardless of how many guiding principles were used. This is not surprising, as critical guiding principles, such as clarity, transparency, compassion and empathy, and timeliness, were not widely incorporated in their posts. Clarity involves using clear language to target and tailor communications for various audiences, which enhances understanding and relevance of the information [15,44,45], increases attention to the message, and increases the likelihood that the message will be shared [33]. All crisis communication posts should incorporate clarity to ensure it is understood and relevant to different public health audiences.

Transparency is rated as the most important strategy in maintaining trust during pandemics [44]; however, it was only used in approximately 4% of posts by Healthy Canadians. In previous research, three-quarters of Canadians reported wanting information about the uncertainty regarding COVID-19 [46]. Transparency demonstrates the effectiveness of public health strategies, allows for the public to assess their risk [44], and cues other actors to anticipate changing recommendations [47]. Transparency is vital for all crisis communication, as it builds relationships and trust with other actors [44].

Compassion and empathy validate the emotions of the public and aid in demonstrating trustworthiness through expressing a willingness to act to avoid future tragedy and loss [34,36,38]. Uncertainty regarding emerging infectious diseases causes fear and anxiety among the public [48]. Compassion and empathy were used in less than 5% of public health messaging missing a key opportunity to demonstrate trustworthiness.

Timeliness has also been shown to build and maintain trust during a pandemic [4] but was seldom used by Healthy Canadians. A lack of timely information, as seen in public health posts, creates an information gap that makes the public more vulnerable to mis- and disinformation [49]. Public health should be clear and timely about what is known and unknown to debunk mis- and disinformation during the COVID-19 infodemic [50].

### 4.2. Lessons Learned: News Media Should Expand the Focus of Their Messaging to Increase Message Acceptance

News media play a critical role in shaping the public’s perception of risk and are a key actor in crisis communication and the response [51,52]. News media serve to provide information that appeals to their audiences by providing alternative perspectives, interpretations, and points of view [9]. Although the media is not a direct part of the public health system, they play a critical role in health education and communication through amplification of public health messages [51].

Media coverage of COVID-19 around the globe dominated headlines in early 2020, with coverage ebbing and flowing with the crisis communication cycle of periods of new information followed by periods with somewhat less new information [53]. One study found that media coverage of the COVID-19 pandemic was driving the public’s attention rather than the pandemic’s progression, which has also been found in other contexts such as H1N1, Zika virus, and influenza [54]. These findings further demonstrate the critical role the media play in shaping the public’s attention, risk perception, knowledge, and awareness of public health emergencies [54]. The media’s framing of crisis messages and the timing of the delivery play a critical role in driving the public’s attention and adoption of risk protective measures [54]. The emotional response to news coverage contributes to the public’s pandemic response [55], and the overall higher negative sentiment towards news media found in this study is of concern.

Our study found the news media primarily shared messages that focused on the pandemic *situation*, such as deaths and cases, but lacked sufficient posts regarding reliable *resources* and directive *actions* that the public should implement to protect themselves from illness or injury. Messages solely focused on the situation saw the highest negative-to-positive comment ratio, which may be due to the invocation of fear without also providing coping actions [56]. While it is important that the media provides information about numbers, such as cases and deaths regarding the situation to inform risk perception [56], content promoting empowerment and efficacy around recommendations are also required to encourage the acceptance of the messages and adoption of risk protective measures [4,34,47,57]. Messaging that focuses on *actions* and *resources* that convey solutions increase the likelihood of individuals complying with recommended behaviours [34,47] and result in more positive sentiment towards the news stories.

### 4.3. Recommendation: Build Trust among Actors through Effective Crisis Communication and Use of the Guiding Principles

One key outcome integral to each of the guiding principles is trust. However, guiding principles used on their own are not sufficient to build trust and promote action and must be used together as relevant to the message content. Key characteristics of crisis communication shown to enhance or maintain trust through impacting the perception of trustworthiness include transparency, timeliness, empathy, correction of misinformation, and clarity [4,34,47,58]. Trust can be developed and maintained through focusing on demonstrating trustworthiness using, in combination, the critical guiding principles [14]. Effective communication demonstrates public health’s trustworthiness by showing other actors that they are acting with integrity and benevolence and have the ability to do so [7,59]. In the short-term, the benefits of building trust with the public increase the uptake of risk protective measures, including vaccination [44]. In the longer term, building and maintaining trust is vital to public health successfully promoting and protecting health and managing future acute or chronic emergencies.

Public health must build positive and trusting relationships with news media with the mutual goal of consistent and accurate media coverage of pandemics [9]. Building trust with the media through the use of guiding principles that demonstrate public health’s trustworthiness provides the media with timely, balanced, and clear information that allows them to better amplify the correct messages. Effective communication is a vital component of strategic crisis communication, where trustworthiness can be demonstrated to build and maintain trust among actors in the crisis arena.

### 4.4. Recommendation: Monitor Social Media Sub-Arenas to Assess Message Acceptance

Overall, the quality and relevance of crisis communication should be assessed through listening, monitoring, and engaging in conversation with crisis publics on the various sub-arenas [16,17]. Public health and news media should monitor comments on posts made by the public within social media sub-arenas to assess if messaging is responded to positively. The news media had a slightly larger proportion of comments with negative sentiment when compared to Healthy Canadians. Globally, English news headlines during the first wave of COVID-19 focused on situational information (e.g., death, hospital, and cases), which evoked 52% negative sentiment compared to 30% positive [60]. Negative sentiment can have very negative implications on social and economic well-being, which has also been seen in past pandemics [60]. Sentiment analysis provides the ability to quickly understand the publics’ emotional response to crisis messages and adjust accordingly to increased acceptance and intended effects of the messages. Both public health and news media can use the guiding principles consistently to increase positive sentiment and build trust among followers.

### 4.5. Limitations

The detailed timeline published by CMAJ News [42], used to assess timeliness, may not have been fully comprehensive, leading to the underestimation of *timeliness* among the Facebook posts. In addition, timeliness within one sub-arena does not actually reflect timeliness in the full crisis communication arena.

Facebook was also a source of limitations as automated data extraction comes with many barriers, which resulted in the research team manually collecting publicly available data. Given the manual data collection, limited sources and posts were collected, amounting to a relatively small data set compared to those collected by web-scraping algorithms, application programming interfaces, or other automated means. Additionally, while Facebook is the most popular social networking site in Canada, the results cannot be generalized beyond understanding how the included sources engaged in crisis communication on Facebook in Canada.

We conducted sentiment analysis on comments which has several limitations. Sentiment analysis is optimized for short, informal pieces of text. SentiStrength is a powerful and fast form of natural language processing with close to human accuracy [31]. However, language is complex and grammatical nuances, cultural variations, tone, slang, and forms of humour, such as sarcasm, are difficult to assess by a computer program [31]. An increase in the accuracy of the results can be achieved by completing a sentiment analysis on larger amounts of data to decrease the error the various nuances described above on can have on sentiment [31].

### 4.6. Future Research Directions

Researchers should investigate the relative importance of each guiding principle for crisis communication via social media and the relationship to sentiment with larger data sets including more actors across public health, government, and news media and in other communication channels. The relationship between the message content, or topic, and its relationship to trust should be further explored. A focus on how to effectively combine the message content with guiding principles to maintain trust should be explored in future research.

## 5. Conclusions

Public trust is vital to controlling the COVID-19 pandemic and failing to provide effective crisis communication erodes that trust. Guiding principles that increase the effectiveness of crisis communication were not widely or consistently found in Facebook posts by Healthy Canadians, CTV News, and CBC News. Public health must provide news media and the public with crisis communication that reflects the guiding principles to ensure understanding and to maintain trust, which increases the acceptance of and compliance with risk protective behaviours. During such uncertain and unpredictable times as a global pandemic, news media and public health officials must provide clear and direct messages empowering the public to take steps to protect themselves based on evidence-informed recommendations.

## Figures and Tables

**Table 3:** Number and percentage of Facebook posts incorporating seven guiding principles for social media crisis communication used by Healthy Canadians, CBC News, and CTV News.

	Call to Action ^1^	Clarity ^1^	Compassion ^1^	Conversational Tone ^1^	Correction of Misinformation ^1^	Timeliness ^1^	Transparency ^1^
	*n* (%)	*n* (%)	*n* (%)	*n* (%)	*n* (%)	*n* (%)	*n* (%)
Healthy Canadians	110	23	5	101	4	7	4
	(98.21%)	(20.53%)	(4.46%)	(90.18%)	(3.57%)	(6.35%)	(3.57%)
CBC News	156	27	3	42	2	38	3
	(99.36%)	(17.20%)	(1.91%)	(26.75%)	(1.27%)	(24.20%)	(1.91%)
CTV News	156	27	3	42	2	38	3
	(92.31%)	(15.98%)	(1.78%)	(24.85%)	(1.18%)	(22.49%)	(1.78%)

^1^ The guiding principles are not mutually exclusive, meaning multiple can be coded per post.

**Table 4:** Percentage of Facebook posts combing seven guiding principles for Healthy Canadians, CBC News, and CTV News.

Source	0, 5, 6, 7 Guiding Principles Used	1 Guiding Principle Used	2 Guiding Principles Used	3 Guiding Principles Used	4 Guiding Principles Used
		%	%	%	%
Healthy Canadians	0	7.14%	61.61%	28.57%	12.42%
CBC News	0	40.76%	43.95%	14.65%	2.68%
CTV News	0	40.24%	46.75%	12.42%	0

**Table 5:** Number and percentage of Facebook posts incorporating topics for Healthy Canadians, CBC News, and CTV News.

Source	Action ^1^ *n* (%)	Resource ^1^ *n* (%)	Situation ^1^ *n* (%)
Healthy Canadians	61 (54.46%)	100 (89.29%)	29 (25.89%)
CBC News	5 (3.18%)	22 (14.01%)	144 (91.72%)
CTV News	1 (0.59%)	31 (18.34%)	157 (92.30%)

^1^ Topics are not mutually exclusive, meaning multiple can be coded per post.

**Table 6:** Summary of sentiment for Healthy Canadians, CBC News, and CTV News.

Source	Total Positive Sentiment *n* (%)	Total Neutral Sentiment *n* (%)	Total Negative Sentiment *n* (%)
Healthy Canadians	717 (32.43%)	721 (32.1%)	773 (34.96%)
CBC News	3196 (27.66%)	3611 (31.25%)	4747 (41.10 %)
CTV News	3440 (26.60%)	4238 (32.78%)	5252 (40.62%)

*X*^2^ = 45.975 on 4 degrees of freedom, *p* < 0.05.

**Table 7:** Summary of Facebook comment sentiment for Healthy Canadians, CBC News, and CTV News combined by guiding principle.

Guiding Principles	Positive	Neutral	Negative
Call to Action	7352	8572	10,773
Clarity	980	1223	1557
Compassion	92	110	171
Conversational Tone	1789	2118	2488
Correction of Misinformation	181	202	376
Timeliness	1706	2145	2446
Transparency	128	164	206

*X*^2^ = 51.087 on 12 degrees of freedom, *p* < 0.05.

**Table 8:** Summary of Facebook posts, comments, and comment sentiment by topic for Healthy Canadians, CBC News, and CTV News combined.

Topic	Total Posts	Total Comments	Positive	Neutral	Negative
Action	67	1908	606	654	648
Resource	153	4059	1177	1312	1570
Situation	330	24,333	6663	7763	9907

*X*^2^ = 39.709 on 4 degrees of freedom, *p* < 0.05.

## Data Availability

The data presented in this study are available on request from the corresponding author.
